# Chinese Herbal Medicines as an Adjunctive Therapy for Unresectable Pancreatic Cancer: A Systematic Review and Meta-Analysis

**DOI:** 10.1155/2015/350730

**Published:** 2015-11-23

**Authors:** Bin Li, Run Gan, Quanjun Yang, Jinlu Huang, Pengguo Chen, Lili Wan, Cheng Guo

**Affiliations:** ^1^Department of Pharmacy, Shanghai Jiao Tong University Affiliated Sixth People's Hospital, Shanghai 200233, China; ^2^Department of Integrative Oncology, Benxi Central Hospital of China Medical University, Benxi, Liaoning 11700, China; ^3^Postgraduate School, Shanghai University of Traditional Chinese Medicine, Shanghai 201203, China

## Abstract

Pancreatic cancer is a common malignancy with a high mortality. Most patients present clinically with advanced pancreatic cancer. Moreover, the effect of radiotherapy or chemotherapy is limited. Complementary and alternative medicines represent exciting adjunctive therapies. In this study, we ascertained the beneficial and adverse effects of Chinese herbal medicine (CHM) in combination with conventional therapy for inoperable pancreatic cancer by using meta-analysis methods for controlled clinical trials. We extracted data for studies searched from six electronic databases that were searched and also assessed the methodological quality of the included studies. We evaluated the following outcome measures: 6-month and 1-year survival rate, objective response rate, disease control rate, quality of life, and adverse effects. The final analysis showed CHM is a promising strategy as an adjunctive therapy to treat advanced or inoperable pancreatic cancer and that CHM in combination with conventional therapy is a promising strategy for resistant disease. However, convincing evidence must be obtained and confirmed by high-quality trials in future studies.

## 1. Introduction

Pancreatic cancer is one of common malignancies and is frequent worldwide. Moreover, pancreatic cancer represents a highly lethal disease due to its high rate of malignancy and invasion as well as its asymptomatic development. Reports from previous work have indicated that pancreatic cancer is the eighth leading cause of death and the ninth leading cause of death from cancer in men and in women worldwide, respectively [1]. Patients with pancreatic cancer exhibit poor survival; only 5% patients will survive 5 years after diagnosis [2]. In China, pancreatic cancer exhibits the seventh highest morbidity rate and the sixth highest mortality rate from cancer according to the 2012 oncology annals [3].

Currently, surgical resection is the optimal and only potentially curable treatment for patients with pancreatic cancer. However, most patients exhibit advanced disease; only 15–20% of patients are considered candidates for surgical resection [4] and 10–15% patients are resectable at diagnosis [5]. Therefore, radiotherapy, chemotherapy, and an aggressive combination are considered the primary and most meaningful therapy options in advanced pancreatic adenocarcinoma. Of all chemotherapies, 5-fluorouracil- (5-FU-) based regimens [6] and gemcitabine-based regimens [7], have been confirmed to exhibit some clinical effects. Promising medicines such as albumin-bound paclitaxel [8] and old medicines, such as irinotecan and oxaliplatin, have been evaluated for clinical effects in clinical trials in locally advanced and metastatic pancreatic cancer patients. Radiotherapy exhibits a substantial advantage with respect to local control and improving the resectability rate after downstaging; therefore, a combination of radiotherapy and chemotherapy should theoretically be regarded as the most effective strategy in locally advanced pancreatic cancer. However, randomized trials to date have yielded conflicting results regarding the survival benefits of CRT in unresectable pancreatic cancer [5]. In addition, specific radiotherapy modalities, including intensity modulated radiotherapy, TOMO, and stereotactic radiotherapy, have been applied to pancreatic cancer treatment and partially improve survival outcomes. Nonetheless, overall survival is unsatisfactory compared with tumors in other sites, and the toxicity of radiotherapy is remarkable. Therefore, additional therapies for this stubborn and deadly disease are critical. Complementary and alternative medicines can perhaps benefit pancreatic cancer patients as an adjunctive therapy.

Of all complementary and alternative medicines, Chinese herbal medicine (CHM) has become increasing prominent and popular in patients with advanced cancer due to its efficacy and low toxicity [9]. A survey of studies deposited in the PubMed database from 1960 to 2013 indicates that more than 450 papers on herbal medicines appeared in the area of cancer prevention and therapy [10]. The rise of published papers related to cancer in recent decades reveals that this small research field of cancer treatment with CHM has undergone a booming development. Moreover, evidence from this literature suggests that traditional Chinese medicine (TCM) can improve the quality of life (QOL) and progression-free survival (PFS) of advanced non-small-cell lung cancer (NSCLC) patients as maintenance therapy [11], increase the efficacy and decrease toxicity in non-small-cell lung cancer patients as an adjunctive therapy [12], and provide a compelling therapeutic option in hepatocellular carcinoma as monotherapy [13]. Though Lu et al. [14] studied the role of TCM in advanced pancreatic cancer by meta-analysis in 2004, the study was limited by the literature included, unclear outcome measures, and language, especially that the adverse effects were scarce. Therefore, we performed this comprehensive meta-analysis and systematic review. The aim of this study is to ascertain the efficacy and adverse effects (AEs) of CHM as an adjunctive therapy for unresectable advanced pancreatic cancer.

## 2. Methods

### 2.1. Search Strategy

We searched related literature from the following major Chinese or English language electronic databases: PUBMED (up to April 2015), Embase (1980–April 2015), Cochrane library, Chinese National Knowledge Infrastructure (CNKI, 1978–April 2015), Wanfang database (1994–April 2015), VIP database (1989–April 2015), and China Biology Medicine disc (CBM disc, 1978–April 2015). Meanwhile, we performed searches using various combinations of terms: pancreatic cancer; pancreatic carcinoma; pancreatic neoplasia; traditional Chinese medicine; CHM; treatment; and clinical trial. In addition, reviews related to this topic were searched to find relevant data. Furthermore, the references from the retrieved studies were scanned carefully for additional relevant studies. When the same trial was reported by different journals or at a different time, we included the most recent study or the one with overall outcome measures. When the same trial was presented as full context or abstract, only the full article was selected to be evaluated.

### 2.2. Study Selection and Outcome Measures

In this meta-analysis, inclusion criteria are in accordance with the following: (1) the patients have a definite diagnosis by either histopathology or imaging examination, such as computerized tomography (CT) or magnetic resonance imaging (MRI); (2) the trial is a clinical, randomized, controlled, and prospective trial; (3) the patients of each study are divided into at least two arms, and the intervention of one arm is chemotherapy, radiotherapy, transcatheter arterial chemotherapy, high intensity focused ultrasound, or the combination of two methods, whereas the intervention in the other arm is the intervention measure of the control group plus Chinese herbal medicine; (4) evaluation of the effect is one of the primary outcome measurements; and (5) the patients included in the studies are adults aged between 18 and 70 years. Exclusion criteria of this meta-analysis were as follows: (1) the clinical trials which are not in accordance with inclusion criteria; (2) the studies which included pregnant or breastfeeding patients or those with another malignancy; (3) the study which is not original research but represents a review or anecdotal report; (4) duplicate studies; and (5) reports in which outcome measures are not extracted.

In addition, outcome measures included primary and secondary indices. The 6-month survival rate (SR), 1-year SR, and objective response rate (ORR) were regarded as the main outcome measures, whereas the disease control rate (DCR), quality of life (QOL), clinical benefit response (CBR), and adverse effects (AEs) were considered secondary indices of evaluation. Moreover, data related to AEs, including different grades of leukopenia and thrombocytopenia and severe grades of nausea and vomiting, were pooled to analyze the effect of CHM on overall toxicity.

### 2.3. Data Extraction and Quality Assessment

In this study, two investigators (Run Gan and Bin Li) reviewed the eligible studies and extracted the data independently. When disagreement existed, a third investigator (Cheng Guo) took part in the discussion and reached consensus for all items. The following data were collected from each article: (1) basic information such as language, year of publication, and first author's name; (2) characteristics including the total number of patients, sample size of each group, age, sex, and disease stage; (3) information on study design, such as randomization method, inclusion criterion, primary end points, and intervention medicines; and (4) information concerning outcome measures, including 1-year SR, ORR, DCR, QOL, and AEs. If the outcome measures were showed as other values, we extracted the pertinent information from the reports. The available information extracted was recorded using a data collection form and saved into electronic databases. Moreover, the quality of the included studies was evaluated by the quantitative 5-point Jadad scale, which contains the report of methods and the results of the studies [15].

### 2.4. Data Analysis

The analysis was undertaken on an intention-to-treat basis. In the statistical analysis, count data and measurement data were presented as MD or RR, respectively. All CIs exhibited two-sided probability coverage of 95%. Heterogeneity among the trials was tested by *χ* ^2^-based  *Q*-statistics [16], and the value of *I* ^2^ was used to determine the presence of heterogeneity. If *P* < 0.01 or *I* ^2^ > 50%, heterogeneity was considered statistically significant; otherwise it was determined that there be no heterogeneity. If there was heterogeneity, the data were analyzed using a random-effect model; otherwise, the data were processed using a fixed-effect model in the absence of heterogeneity. Publication bias was examined through a funnel plot and statistical tests, including the Begg or Egger tests [17, 18]. All statistical calculations were performed using Review Manager 5.3 software (The Nordic Cochrane Centre, Copenhagen, Denmark) and Stata 12.0 software (Stata Corporation, College Station, TX, USA).

### 2.5. Sensitivity Analysis

In this study, sensitivity analysis was performed to verify the robust and reliable results from our study. We completed the analysis by excluding some trials which had a quality score of 1.

## 3. Results

### 3.1. Quantity and Quality of the Literature

**Figure 1:**
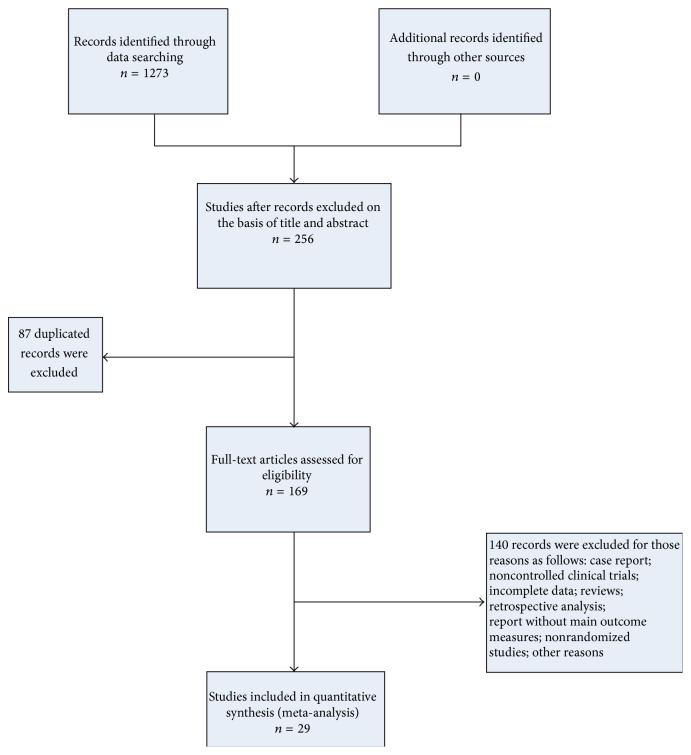
Flow chart of study selection.

In this study, 1273 articles were originally identified from six electronic databases by the search strategies described in Section 2. After duplicated studies and reports unrelated to clinical study of pancreatic cancer were excluded by title and abstract, 172 full-text papers were screened carefully. One hundred forty-three records were excluded for the following reasons: experimental reports, retrospective study, semirandomized trial, noncontrolled trial, duplicates, primary outcome measures unable to be extracted, or other reasons. After exclusion, 29 studies were eligible for inclusion in this meta-analysis (Figure 1).

The overview of the 29 papers included is indicated in Table 1. Of those clinical trials, 27 studies were published in Chinese language and 2 studies [19, 20] were reported in English language. All studies were performed in China expect for 1 [19] in Japan, and the studies involved a total of 1808 patients with advanced pancreatic cancer. In addition, there were only three studies with Jadad score ≥3 [20–22]. Meanwhile, all studies exhibited comparable baseline patient characteristics, including age, gender, and stage, and there were no significant differences among them.

### 3.2. Six-Month and One-Year Survival Rate

**Figure 2:**
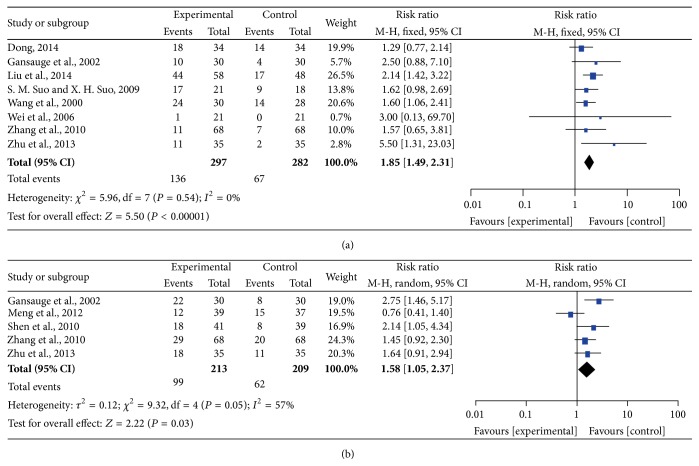
Forest plots of 6-month SR and 1-year SR. (a) represents the fixed-effects model of the risk ratio (95% CI) of 1-year SR associated with CHM-containing versus non-CHM-containing regimens; (b) represents the random-effects model of the risk ratio (95% CI) of 6-month SR associated with CHM-containing versus non-CHM-containing regimens.

Five studies showed 6-month SR and eight studies reported 1-year SR, and the analysis of the pooled results is presented by forest plot in Figure 2. There was no significant heterogeneity among the studies (*I* ^2^ = 0%, *P* = 0.54) for 1-year SR; therefore, we performed the analysis using a fixed-effects model; however, there was significant heterogeneity among the trials (*I* ^2^ = 57%, *P* = 0.05) for 6-month SR; therefore, the pooled RR was analyzed using a random-effects model. The pooled RRs of 6-month SR and 1-year SR are 1.58 (95% CI = 1.05–2.37, *P* = 0.03) and 1.85 (95% CI = 1.49–2.31, *P* < 0.00001) in the CHM-containing group, respectively, and clearly indicated that treatment with CHM-containing regimens significantly improves 1-year SR compared with the non-CHM-containing regimens.

### 3.3. Objective Response Rate

**Figure 3:**
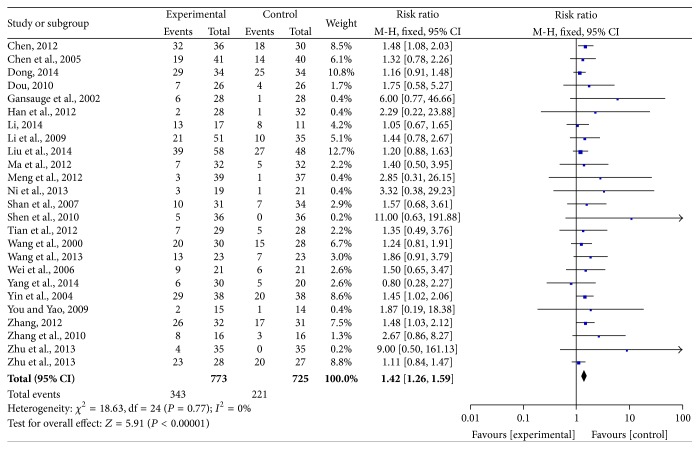
Forest plot of the fixed-effects model of the risk ratio (95% CI) of ORR associated with CHM-containing versus non-CHM-containing regimens.

Twenty-five trials exhibited ORR as an outcome measure. The pooled RR for ORR revealed that there was a remarkable improvement for CHM-containing treatment yielding a RR of 1.42 (95% CI = 1.26–1.59, *P* < 0.00001). There was no significant heterogeneity among the trials (*I* ^2^ = 0%, *P* = 0.77); therefore, the pooled RR was performed using a fixed-effects model (Figure 3).

### 3.4. Disease Control Rate

**Figure 4:**
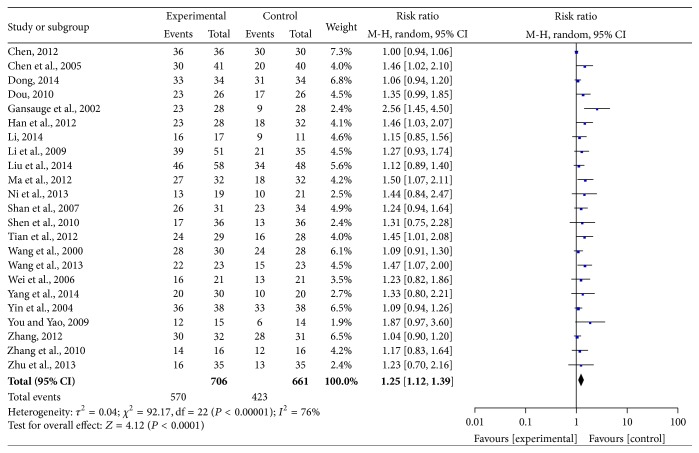
Forest plot of the random-effects model of the risk ratio (95% CI) of DCR associated with CHM-containing versus non-CHM-containing regimens.

DCR could be definitively extracted from twenty-three reports. The pooled RR for ORR demonstrated that there was a significant improvement in CHM-containing treatments, yielding RR of 1.25 (95% CI = 1.12–1.39, *P* < 0.0001). There was significant heterogeneity among the trials (*I* ^2^ = 76%, *P* < 0.00001); therefore, the pooled RR was analyzed using a random-effects model (Figure 4).

### 3.5. Clinical Benefit and Quality of Life

**Figure 5:**
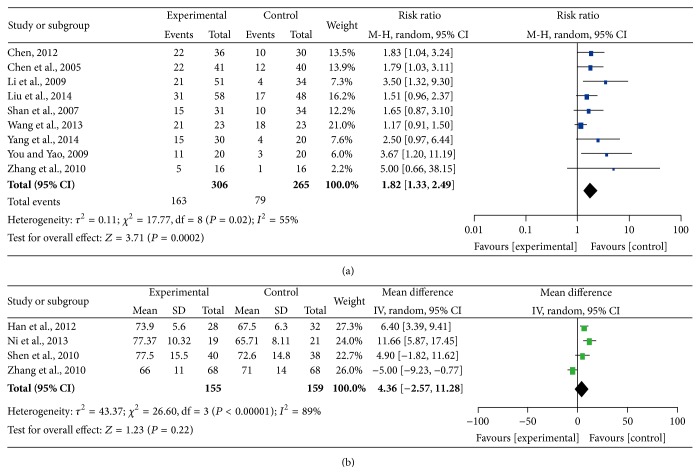
Forest plots of the impact on quality of life. (a) represents the random-effects model of the risk ratio (95% CI) of quality of life associated with CHM-containing versus non-CHM-containing regimens by expression data; (b) represents the fixed-effects model of the mean difference (95% CI) in quality of life associated with CHM-containing versus non-CHM-containing regimens by expression data.

Thirteen trials reported improvement of QOL; however, this outcome was measured in different manners. Nine studies analyzed QOL by using specific scores (count data), and four studies reported the results as the number of patients reporting improvements (measurement data). Therefore, we performed a pooled analysis by using the expression of RR and WD, respectively. There was significant heterogeneity among the trials (*I* ^2^ = 55%, *P* = 0.02; *I* ^2^ = 89%, *P* < 0.00001); therefore, the pooled RR was analyzed using a random-effects model. The pooled RR for QOL demonstrated that there was an improvement for CHM-containing treatments, giving a RR of 1.25 (95% CI = 1.12–1.39, *P* = 0.0002) for the measurement data; however, the pooled MD for QOL revealed that there was no improvement for CHM-containing treatment, with an MD of 4.36 (95% CI = −2.57–11.28, *P* = 0.22) for count data (Figure 5).

**Figure 6:**
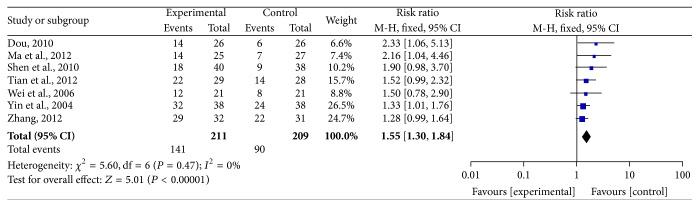
Forest plot of the fixed-effects model of the risk ratio (95% CI) of CBR associated with CHM-containing versus non-CHM-containing regimens.

Seven trials reported CBR and were included in the analysis (Figure 6). The results are presented in Figure 6. CBR in the pooled trials indicated a significant rise in CHM-containing compared to non-CHM-containing treatments, yielding a RR of 1.55 (95% CI = 1.30–1.84, *P* < 0.00001). We performed this analysis using a fixed-effects model because there was no significant heterogeneity among the trials (*I* ^2^ = 0%, *P* = 0.47).

### 3.6. Adverse Effects

**Figure 7:**
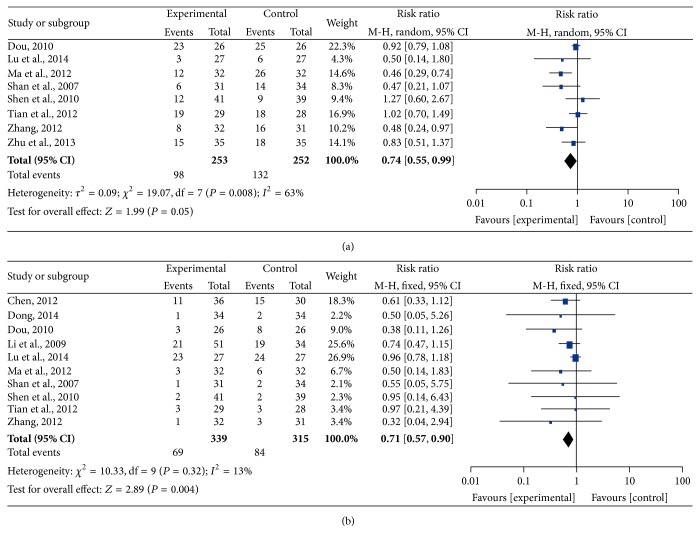
Forest plots of the impact on leukopenia. (a) represents the random-effects model of the risk ratio (95% CI) of grade I–IV leukopenia associated with CHM-containing versus non-CHM-containing regimens; (b) represents the fixed-effects model of the risk ratio (95% CI) of grade III-IV leukopenia associated with CHM-containing versus non-CHM-containing regimens.

**Figure 8:**
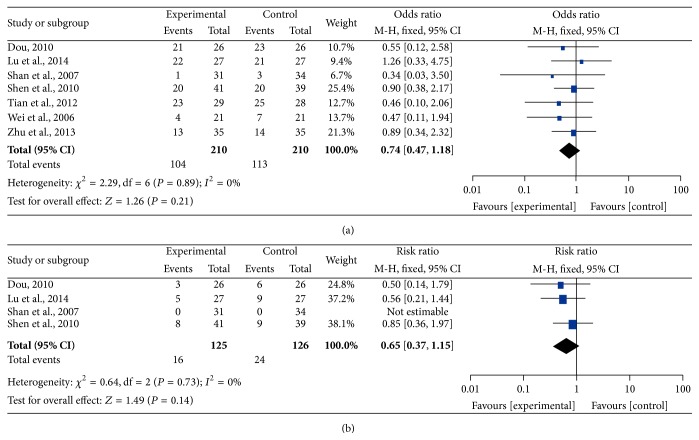
Forest plots of the impact on thrombocytopenia. (a) represents the fixed-effects model of the risk ratio (95% CI) of grade I–IV thrombocytopenia associated with CHM-containing versus non-CHM-containing regimens; (b) represents the fixed-effects model of the risk ratio (95% CI) of grade III-IV thrombocytopenia associated with CHM-containing versus non-CHM-containing regimens.

**Figure 9:**
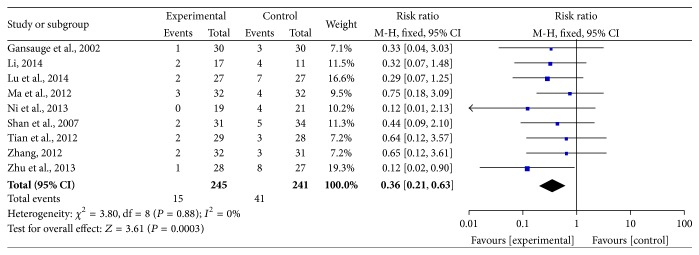
Forest plot of the fixed-effects model of the risk ratio (95% CI) of grade III-IV nausea and vomiting associated with CHM-containing versus non-CHM-containing regimens.

Bone marrow suppression and gastrointestinal reactions were frequent symptoms in the treatment of malignant tumors; therefore, the data concerning leukopenia and thrombocytopenia were pooled for the analysis of myelosuppression (Figures 7 and 8), and the incidence of severe nausea and vomiting was pooled as gastrointestinal reaction (Figure 9). All data were pooled using a fixed-effects model because of the absence of heterogeneity exclusive of grade I–IV leukocytopenia (grade III-IV nausea and vomiting: *I* ^2^ = 0%, *P* = 0.88; grade III-IV leukocytopenia: *I* ^2^ = 13%, *P* = 0.32; grade I–IV thrombocytopenia: *I* ^2^ = 0%, *P* = 0.89; grade III-IV thrombocytopenia: *I* ^2^ = 0%, *P* = 0.73), and the data for grade I–IV leukocytopenia were pooled by using a random-effects model for the presence of heterogeneity (*I* ^2^ = 63%, *P* = 0.008). The pooled RRs were 0.36 (95% CI = 0.21–0.63, *P* = 0.0003) and 0.71 (95% CI = 0.57–0.90, *P* = 0.004) for the incidence of gastrointestinal reaction and grade III-IV leukopenia, respectively, which demonstrated that the rates of AEs for CHM-containing treatments were remarkably less than for non-CHM-containing regimens. Meanwhile, the remainder of pooled RR values were 0.74 (95% CI = 0.55–0.99, *P* = 0.05), 0.74 (95% CI = 0.47–1.18, *P* = 0.21), and 0.65 (95% CI = 0.37–1.15, *P* = 0.14) for grade I–IV leukopenia, grade I–IV thrombocytopenia, and grade III-IV thrombocytopenia, respectively, which indicated that there was no obvious difference in these AEs compared with the control group.

### 3.7. Sensitivity Analysis

When those literatures with a quality score of 1 were excluded, the sensitivity analysis indicated that the pooled RR and 95% CI for 1-year SR, ORR, DCR, and gastrointestinal reaction were only norminally different from values calculated for the entire data. The results were showed in Table 2.

Though the sensitivity analysis is completed, we can find that the study was not very sensitive to study quality; meanwhile, it also showed that the results of our study were reliable and verifiable.

### 3.8. Publication Bias

**Figure 10:**
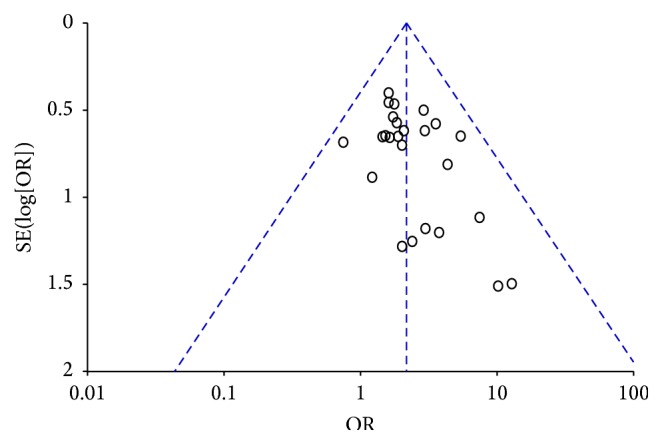
Asymmetric funnel plot of the ORR in the included studies.

Funnel plots and Egger's test were performed to identify potential publication bias among the included studies. The shapes of the funnel plots revealed some evidence of obvious asymmetry, and the representative funnel plot for ORR is presented in Figure 10. Subsequently, Egger's test was used to provide statistical evidence of funnel plot symmetry. The results also revealed some evidence of publication bias (ORR: *P* = 0.001; DCR: *P* = 0.000; QOL: *P* = 0.000; CBR: *P* = 0.006; grade III-IV leukopenia: *P* = 0.019).

### 3.9. Analysis of Chinese Herbal Medicine Characteristic

**Figure 11:**
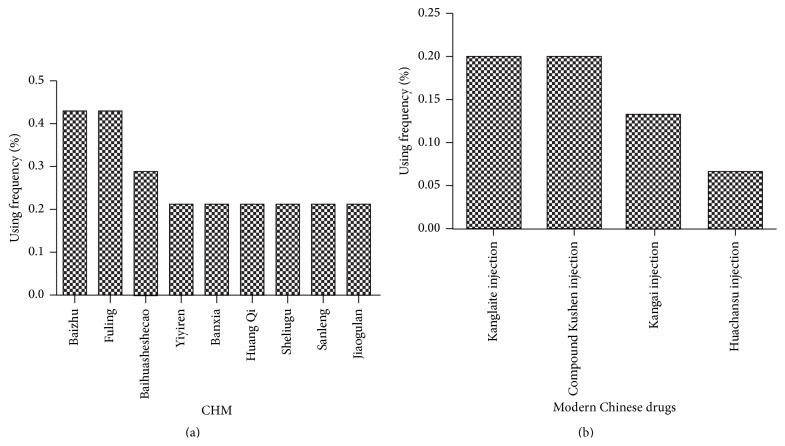
Frequency of use of CHM. (a) indicates the percentage of polyherbal medicines that include traditional CHM, whereas (b) indicates the percentage of herbal medicines that processed into modern CHM.

In the included studies, 15 were designed using active ingredients of CHM that were processed into modern preparation such as injection or capsule. The remaining trials were designed using traditional decoction in combination with the same treatment as the control intervention. CHMs in order of the frequency of use were as follows: Baizhu (Rhizoma Atractylodis Macrocephalae, 6/14), Fuling (*Poria cocos*, 6/14), Baihuasheshecao (*Hedyotis diffusa*, 4/14), Yiyiren (Semen Coicis, 3/14), Banxia (Rhizoma Pinelliae, 3/14), Huang Qi (Radix Astragali, 3/14), Sheliugu (Rhizoma Amorphophalli, 3/14), Sanleng (Rhizoma sparganii, 3/14), and Jiaogulan (*Gynostemma pentaphyllum*, 3/14). Modern Materia medica preparations were mainly used, which were derived from CHM and were utilized as follows: Kanglaite injection (3/15), Kangai injection (2/15), compound Kushen injection (3/15), and Huachansu Injection (1/15). The frequency of use is indicated in Figure 11.

## 4. Discussion

TCM has increasingly drawn a wider range of interest as a complementary and alternative therapy among international cancer research studies because it can increase efficacy and decrease toxicity when combined with radiotherapy and chemotherapy. Furthermore, the integration of palliative care in cancer patients has become standard oncology practice when a patient is diagnosed with metastatic or advanced cancer according to NCCN clinical practice guidelines [48, 49]. In China, TCM has a longstanding history and is deeply embedded in rural and urban populations as a measure of palliative care. To our excitement, TCM has also been accepted into Chinese clinical practice guidelines in the treatment of pancreatic cancer [50]. Recent reported studies have demonstrated that 90% of Chinese patients with cancer have received diverse TCM treatments during their treatment regimen [10]. Preclinical studies have demonstrated that CHM can suppress tumor proliferation and metastasis. For example, the famous Qingyi Huaji formula, which was found and established from the cancer center of Fudan University in China, can inhibit the growth of liver metastasis from pancreatic cancer in nude mice [51], inhibit the cell cycle in pancreatic cancer CFPAC-1 cells [52], and inhibit pancreatic cancer cell invasion and metastasis in part by reversing tumor-supporting inflammation [53]. Though CHM has multiple complicated components and probable AEs, its application has been widely embraced in clinical practice, especially throughout China. This phenomenon is attributed to the fact that the origin and development of TCM are intricately entwined with Chinese history, culture, economy, and politics and that its compelling efficacy has been attested. The present study has revealed that CHM can increase the role of antitumor therapies and improve PS or QOL in pancreatic cancer patients, which will provide more evidence to promote the application of CHM in China as well as to gain worldwide approval and benefit for pancreatic cancer patients.

The increase in overall survival remains the still focus of treatment of cancer patients, and the efficacy of antitumor treatment is typically evaluated by observing effects on survival. Prior to our meta-analysis [15], a meta-analysis was published in Chinese, but there was no evaluation associated with survival time, ORR, QOL, and AEs. In this study, more than fivefold the number of studies were included in the pooled analysis. The 6-month SR and 1-year SR of CHM-containing regimens are clearly increased compared with non-CHM-containing schemes in patients with advanced pancreatic cancer by using random-effects and fixed-effects model respectively, which suggests that CHM contributes to prolonged survival. Unfortunately, only eight (32.14%) studies included this important outcome measure. Moreover, the 1-year SR was uneven, and two reports [42, 45] indicated high SR. The reasons for this discrepancy were related to the patients included in the different studies. In addition, the results of this meta-analysis for ORR, DCR, and CBR demonstrated the same advantages observed with respect to survival outcomes. The above data suggest that CHM likely exhibits antitumor role and synergetic effects in combination with other therapies that have been approved worldwide, including in the USA. PHY906 from the traditional Chinese herbal formulation Huang-Qin-Tang has been involved in a series of preclinical studies and clinical research in USA. The treatment appears to be a safe and feasible salvage therapy with treatment with capecitabine plus PHY906 in advanced pancreatic cancer [54]. Improvement of QOL in this meta-analysis has been reported in studies associated with cancer, and the research on PHY906 indicates improvements compared with baseline levels [55]. Our results reveal a partial contradiction for different analyses of QOL. The discrepancy is caused to a great extent by the poor quality of the literature and minor cases of count data. In four studies, one report clearly demonstrated declining QOL. AEs often occur in patients with advanced cancer when chemotherapy, radiotherapy, or the combination of two therapies is administered. Patients are typically tolerant of grade I-II myelosuppression and digestive tract reactions. We analyzed and evaluated severe symptoms of nausea and vomiting, leukopenia, and thrombocytopenia. The results tend to imperfect improvements, which is consistent with previous findings. The grade III-IV symptoms of nausea and vomiting and leukopenia were clearly improved in patients with CHM-containing treatments, although there was no effect on thrombocytopenia.

TCM is based on a completely different theoretical system than Western medicine in which the name of disease is formed by using symptoms of the disease. Pancreatic cancer is attributed to the JiJu and FuLiang symptoms in TCM and has a pathological process that includes deficiency of healthy Qi and excess of evil pathogenic Qi. Therefore, reinforcing the healthy Qi and eliminating excess evil pathogenic Qi, including phlegm, dampness, heat, and stasis toxin, represent the main treatment principles of pancreatic cancer. This study indicated clearly that the application of CHM complied entirely with these principles according to the analysis of CHM frequency. Moreover, certain new reports have indicated that the composition of CHMs includes compounds that regulate immunity function and have antitumor potency in vitro and in vivo, such as ginsenoside Rg3 [56, 57], Astragalus polysaccharides [58], Atractylenolide [59], an ethanol extract of* Hedyotis diffusa* [60], and Bufalin [61].

Meanwhile, in this study, some herbal medicines, which were applied extensively in patients with malignant tumor, were verified to have beneficial role. For example, Baihuasheshecao, an old and well-known traditional Chinese medicine, is composed of abundant chemical ingredients and has antitumor activity. The ethanol extract of* Hedyotis diffusa* Willd. suppresses proliferation and induces apoptosis via IL-6-inducible STAT3 pathway inactivation [62, 63]. Recent literature [64] reported that the novel cyclotides extracted from Baihuasheshecao have anticancer effects and they are potential bioactive ingredients; in addition, methylanthraquinone induces Ca^2+^-mediated apoptosis in human breast cancer cells [65]. In addition, [66] suggested that KLT can suppress growth and induce apoptosis of pancreatic cancer Xenografts by downregulating the expression of phospho-Akt and phospho-mTOR. The current evidences [67] indicated that some antitumor TCMs mainly take their effects on the apoptotic signaling pathway.

Although our study demonstrates favorable outcomes in CHM-containing treatments, the quality of the studies is substandard, and publication bias was indicated by the asymmetric funnel plot. The negative trials results were usually not reported by authors, which was the major reason to the publication bias. In addition, there are other reasons, such as small sample and single central trial. No study was double-blind, and only two trials were single-blind, which leads to a low Jadad grade score. In addition, adequate methods were not specified, and 11 trials were randomized by using random number tables to generate a sequence. The remaining trials also were randomized by using the same methods when we contacted the authors by using email or telephone. Two trials reported the cases that withdrew for various reasons. Usually, studies with Jadad score ≥3 are the most suitable for meta-analysis; however, the poor quality of these reports was most likely caused by irregular reporting as opposed to flaws in the design and execution. What is more, the results are usually more important than the methodology in China, which leads to vague methodology. Therefore, we included all randomized control trials with available main outcome measures. These flaws suggest that such trials should be reported or published with regular expression and terminology worldwide.

In conclusion, the pooled data present compelling evidence that CHM is a promising strategy as an adjunctive therapy in treating unresectable and advanced pancreatic cancer and that TCM in combination with conventional therapy is useful for overcoming this stubborn disease. However, high-quality and precisely evaluated research as well as improvements in the quality of the reported trials, particularly in the descriptions of methodology and study processes, is urgently needed.

## Figures and Tables

**Table 1:** Characteristics of studies in the pooled analysis (*N* = 1808).

Study	Number of participants (T/C )	Sex (females/total)	TNM stage	CHM or CHM formula	Intervention in control group	Outcome assessment	OS (months) or 1-year SR (%)	Duration (week)	Jadad score
Lu et al. 2014 [23]	54 (27/27)	23/57	III-IV	Shenqi Fuzhen injection	Gemcitabine injection, Tegafur Gimeracil potassium capsule	OS, AEs, and symptoms	10.7	6	2

Zhang et al. 2010 [24]	32 (16/16)	13/32	II–IV	Xihuang pill	Gemcitabine injection	ORR, DCR, QOL, AEs, and symptoms	NR	6	2

You and Yao 2009 [25]	40 (20/20)	17/40	III-IV	Fuzhen Hewei decoction	Gemcitabine and oxaliplatin injection	ORR, DCR, QOL, symptoms, and laboratory values	NR	8	2

Dong 2014 [26]	68 (34/34)	33/68	II–IV	Qingyi decoction	Docetaxel and cisplatin injection	ORR, DCR, 1- and 3-year SR, symptoms, APACHE II score, and AEs	52.94%	6	2

Li 2014 [27]	28 (17/11)	12/28	III-IV	Chanchu injection	Gemcitabine injection, radiotherapy	ORR, DCR, and AEs	NR	16–24	2

Wei et al. 2006 [28]	42 (21/21)	13/42	III-IV	Ejiao paste	Gemcitabine, leucovorin, calcium, and fluorouracil injection	ORR, DCR, 1-year SR, CBR, and AEs	4.8%	12	2

Li et al. 2009 [29]	86 (51/35)	none	III-IV	Fuzhenkangai decoction	Leucovorin, calcium, VP-16 cisplatin, and fluorouracil injection	ORR, DCR, QOL, and AEs	9	9	1

Chen 2012 [30]	66 (36/30)	31/66	AS	Compound Kushen injection	Radiotherapy	ORR, DCR, QOL, and AEs	NR	6	1

Dai 2014 [31]	50 (25/25)	23/50	III-IV	Jiedu Huayu Tongfu granules	Gemcitabine injection	ORR, DCR, AEs, symptoms, and laboratory values	NR	12	2

Zhang 2009 [32]	63 (32/31)	25/63	III-IV	Compound Kushen injection	Gamma knife radiosurgery	ORR, DCR, AEs, QOL, CBR, and laboratory values	NR	3	2

Liu et al. 2014 [33]	106 (58/48)	36/106	II–IV	Yiqi Huoxue decoction	Radiotherapy, gemcitabine injection	1 and 2-year SR, ORR, DCR, AEs, and QOL	75.3%	6-7	1

Zhu et al. 2013 [34]	55 (28/27)	24/55	AS	Kanglaite injection	*γ*-SBRT	ORR, AEs, and QOL	NR	6	1

Shan et al. 2007 [35]	65 (31/34)		AS	Kanglaite injection	Fluorouracil and cisplatin injection	ORR, QOL, and AEs	NR	12	1

Zhu et al. 2013 [21]	70 (35/35)	25/70	III-IV	Qinre Huaji decoction	HAI/TACE	1/2- and 1-year SR, ORR, QOL, and AEs	31.43%	16	3

Ni et al. 2013 [36]	40 (19/21)	17/40	II–IV	WD-3 decoction	Gemcitabine, leucovorin, calcium, and fluorouracil injection	ORR, DCR, CBR, QOL, AEs, symptoms, and laboratory values	NR	8	2

Ma et al. 2012 [37]	64 (32/32)	13/64	AS	Kanglaite injection	Gemcitabine injection	ORR, DCR, and AEs	NR	24	1

Han et al. 2012 [38]	65 (31/34)	NR	III-IV	Modified Sinisan decoction	TAI	ORR, AEs, QOL, and symptoms	NR	8	3

Tian et al. 2012 [22]	60 (30/30)	32/60	III-IV	Qingre Jiedu and Huoxue Huayu decoction	Gemcitabine injection	ORR, DCR, CBR, AEs, and laboratory values	NR	8	2

Shen et al. 2010 [39]	80 (41/39)	30/80	III-IV	Qingyi Huaji formula	TAC + 3DCRT	ORR, 1/2-, 1-, 2-, and 3-year SR, OS, CBR, QOL, AEs, and symptoms	9.8%	8	2

Zhang et al. 2010 [40]	136 (68/68)	55/136	AS	Qingyi Huaji decoction	TAC + 3DCRT	1/2- and 1-year SR, QOL, and AEs	16.2%	NR	1

Wang et al. 2013 [41]	46 (23/23)	21/46	AS	Kangai injection	SBRT	ORR, QOL, AEs, and symptoms	NR	3	2

S. M. Suo and X. H. Suo 2009 [42]	39 (21/8)	8/39	AS	Yiqi Huoxue decoction	Radiotherapy, TAI	ORR, 1- and 2-year SR, AEs, and symptoms	82.1%	NR	1

Yang et al. 2014 [43]	50 (30/20)	22/50	NR	Compound Kushen injection	Gemcitabine and oxaliplatin injection	DCR, QOL, and CBR	NR	6–18	1

Yin et al. 2004 [44]	76 (38/38)	28/76	NR	Jinlong capsule	Gamma knife radiosurgery	ORR, CBR, QOL, and AEs	NR	13	2

Wang et al. 2000 [45]	58 (30/28)	15/58	II-III	Yiqi Huoxue decoction	Radiotherapy, TAC	1- and 2-year rate, ORR, symptoms, and AEs	80%	NR	2

Gansauge et al. 2002 [19]	60 (30/30)	19/60	III-IV	NSC-631570	Gemcitabine injection	ORR, DCR, 1/2-, 2/3-, and 1-year SR, AEs, and QOL	32%	12	2

Meng et al. 2012 [20]	76 (39/37)	30/76	NR	Huachansu injection	Gemcitabine injection	ORR, OS, TTP, symptoms, AEs, and 1/2-year SR	5.3	8	3

Chen et al. 2005 [46]	81 (41/40)	36/81	III-IV	Compound Danshen dripping pills	Gemcitabine and cisplatin injection	ORR, DCR, QOL, AEs, and laboratory values	NR	8	1

Dou 2010 [47]	52 (26/26)	27/52	III-IV	Kangai injection	Gemcitabine and cisplatin injection	ORR, CBR, and AEs	NR	8	1

AS: advanced stage; SBRT: stereotactic body radiotherapy; TAI: transcatheter arterial infusion; NR: not reported; HAI: hepatic artery infusion chemotherapy; TAC: transcatheter arterial chemoembolization; 3DCRT: 3-dimensional conformal radiation therapy.

**Table 2:** Sensitivity analysis for all studies versus those studies with score of ≥2.

Outcomes	Meta-analysis for all studies				Meta-analysis for those studies with score of ≥2			
	Number	Total patients (intervention/control groups)	RR (95% CI)	*P* value	Number	Total patients (intervention/control groups)	RR (95% CI)	*P* value
6-month SR	5	422 (213/209)	1.58 (1.05, 2.37)	0.03	4	289 (145/141)	1.63 (0.94, 2.83)	0.08
1-year SR	8	579 (297/282)	1.85 (1.49, 2.31)	0.00001	5	298 (150/148)	1.82 (1.33, 2.49)	0.0002
ORR	25	1498 (773, 725)	1.42 (1.26, 1.59)	0.00001	16	873 (440/433)	1.54 (1.31, 1.80)	0.00001
DCR	23	1367 (706, 661)	1.25 (1.12, 1.39)	0.0001	15	797 (401/396)	1.23 (1.10, 1.37)	0.0003
Gastrointestinal reaction	7	420 (211/209)	1.55 (1.30, 1.84)	0.00001	6	302 (154/148)	0.36 (0.17, 0.73)	0.005
Leukopenia of grades III-IV	10	654 (339/315)	0.71 (0.57, 0.90)	0.004	5	322 (163/159)	0.68 (0.29, 1.58)	0.36
Leukopenia of grades I–IV	8	505 (253/252)	0.74 (0.55/0.99)	0.05	5	324 (164/160)	0.72 (0.45, 1.15)	0.17
Thrombocytopenia of grades I–IV	7	420 (210/210)	0.74 (0.47, 1.18)	0.21	5	303 (153/150)	0.80 (0.48, 1.32)	0.38
